# 无化疗诱导救治Ph阳性急性淋巴细胞白血病危重产妇1例报告并文献复习

**DOI:** 10.3760/cma.j.cn121090-20250519-00235

**Published:** 2025-10

**Authors:** 梦 高, 妍 谢, 子溢 刘, 佩淇 梁, 立民 刘, 杰 殷, 栋 王, 冰 韩, 惠英 仇, 建红 付, 德沛 吴

**Affiliations:** 1 苏州大学附属第一医院血液科ICU，国家血液系统疾病临床医学研究中心，苏州 215006 Hematology Intensive Care Unit, Department of Hematology, the First Affiliated Hospital of Soochow University, National Clinical Research Center for Hematologic Diseases, Suzhou 215006, China; 2 苏州大学附属第一医院妇产科，苏州 215006 Department of Obstetrics & Gynecology, the First Affiliated Hospital of Soochow University, Suzhou 215006, China

## Abstract

报道1例妊娠合并Ph阳性急性淋巴细胞白血病（Ph^+^ALL）危重患者的救治经验。患者为36岁女性，孕28周时确诊Ph^+^ALL，病情迅速恶化，出现弥散性血管内凝血（DIC）、弥漫性肺泡出血（DAH）、脓毒症休克及多器官功能障碍，转入血液科重症监护病房。鉴于患者病情危重且处于妊娠晚期，采用伊马替尼联合地塞米松的“无化疗”诱导方案，同时予气管插管机械通气、连续性肾脏替代治疗（CRRT）、广谱抗感染及大剂量糖皮质激素等综合支持治疗。治疗期间胎儿胎死宫内，经产科干预娩出死胎。经积极救治，患者呼吸衰竭、DIC及DAH逐步改善，白血病达完全缓解，后续完成巩固化疗、嵌合抗原受体T细胞治疗及异基因造血干细胞移植，长期随访显示持续完全分子学缓解。表明对于妊娠合并Ph^+^ALL的危重患者，在强化支持治疗基础上，采用靶向药物联合糖皮质激素的“无化疗”方案安全有效，可为类似患者提供治疗思路。

## Abstract (en)

This report presents the management of a critically ill 36-year-old woman diagnosed with Philadelphia chromosome-positive acute lymphoblastic leukemia（Ph^+^ ALL）at 28 weeks of gestation. The patient rapidly deteriorated, developing disseminated intravascular coagulation（DIC）, diffuse alveolar hemorrhage（DAH）, septic shock, and multi-organ dysfunction, necessitating admission to the hematological intensive care unit. Given her critical condition and advanced pregnancy, a chemotherapy-free induction regimen comprising imatinib and dexamethasone was initiated, alongside comprehensive supportive measures, including mechanical ventilation, continuous renal replacement therapy（CRRT）, broad-spectrum antibiotics, and high-dose corticosteroids. During treatment, intrauterine fetal demise occurred, and a stillborn was delivered following obstetric intervention. With aggressive treatment, the patient's respiratory failure, DIC, and DAH gradually resolved, and she achieved complete remission. She subsequently received consolidation chemotherapy, CAR-T cell therapy, and allogeneic hematopoietic stem cell transplantation, achieving sustained complete molecular remission on long-term follow-up. This case demonstrates that for critically ill pregnant patients with Ph^+^ALL, a chemotherapy-free regimen of targeted therapy and corticosteroids, when combined with intensive supportive care, is a safe and effective approach that may offer a therapeutic option for similar cases.

传统上，Ph染色体阳性急性淋巴细胞白血病（Ph^+^ALL）因对化疗反应差、复发率高而被视为高危亚型，5年总生存率不足10％[1]，近年来，随着BCR::ABL酪氨酸激酶抑制剂（TKI）的临床应用，这一局面得到显著改善。研究证实，第一代TKI伊马替尼联合化疗可使Ph^+^ALL完全缓解率提升至90％以上，5年总生存率提高至50％[2]。更值得关注的是，基于TKI的“无化疗”方案为特殊人群（如老年、体弱或妊娠患者）提供了新的治疗选择。

本文报道1例妊娠晚期合并Ph^+^ALL的危重产妇，患者在诱导治疗初期迅速出现弥散性血管内凝血（DIC）、弥漫性肺泡出血（DAH）、脓毒症休克及多器官功能障碍，病情极为凶险。通过采用伊马替尼联合地塞米松的“无化疗”诱导方案，在血液科重症监护病房（HCU）结合多学科协作的综合支持治疗，患者最终获得完全缓解并长期生存。本案例旨在为类似患者的临床救治提供参考，并探讨“无化疗”方案在妊娠合并Ph^+^ALL危重患者中的应用价值。

## 病例资料

患者，女，36岁，孕4产3，因“停经28周，全身瘀点瘀斑2周”于2021年11月3日从产科转入我院HCU。2021年10月20日实验室检查示：WBC 8.73×10^9^/L，HGB 71 g/L，PLT 10×10^9^/L，外周血涂片见16％原始幼稚细胞。骨髓细胞形态学示增生低下，原始幼稚细胞占26.5％（POX染色阴性），免疫分型提示分析11.3％的幼稚细胞群体，为B淋系表达（CD34^+^、DR^+^、CD10^+^、CD19^+^、CD33^+^、CD79a^+^）。分子遗传学检测显示BCR::ABL融合基因阳性（e1a2转录本，编码P190蛋白），染色体核型为46, XX, dic（7;12）（p11;p11）, t（9;22）（q34;q11）, +Ph[4]/46,XX[6]，确诊为Ph^+^ALL。

入院后予地塞米松预处理，头孢硫脒抗感染、血小板输注及凝血因子补充等支持治疗。11月3日患者病情恶化，出现胸闷、心悸、广泛皮肤瘀斑及肉眼血尿，PLT降至2×10^9^/L，D-二聚体6.09 mg/L，纤维蛋白降解物19.69 mg/L。考虑患者处于晚期妊娠，体质虚弱（28周+5 d），为避免化疗药物对产妇和胎儿的毒性作用，在HCU采用伊马替尼（400 mg/d）联合地塞米松（15 mg/d）的无化疗方案进行诱导治疗。

11月5日凌晨患者出现胸闷、呼吸困难加重，血压降至70/40 mmHg（1 mmHg＝0.133 kPa），听诊双肺湿啰音，胸部影像学提示双肺弥漫性渗出（图1A），立即给予美罗培南+卡泊芬净抗感染、去甲肾上腺素升压、甲泼尼龙240 mg冲击及高流量氧疗后患者症状稍好转。当晚患者病情进一步恶化，出现粉红色泡沫痰，呼吸急促，呼吸频率40次/min，血氧饱和度降至83％，遂行气管插管机械通气（压力控制模式，吸氧浓度70％，吸气压力16 cmH_2_O，频率20次/min，呼气末正压12 cmH_2_O）。支气管肺泡灌洗液呈暗红色血性痰，结合临床表现诊断为DAH。

**图1:**
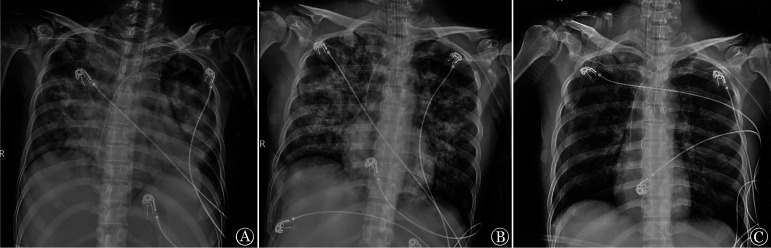
血液科重症监护病房救治过程中X线胸片变化 **A** 2021年11月5日示双肺渗出；**B** 2021年11月6日示双肺渗出较前稍好转；**C** 2021年11月9日示渗出进一步吸收

11月6日患者持续高热（T_max_ 39.5 °C），同时出现宫口已扩张，不排除肺部感染、绒毛膜羊膜炎可能，痰液及咽拭子培养提示洋葱伯克霍尔德菌，血培养阴性，纤支镜肺泡灌洗液高通量病原体测序提示铜绿假单胞菌（序列数10），调整抗生素广覆盖抗感染，同时地塞米松减量至10 mg每日1次。胎心监护显示基线150次/min、变异差，伴频繁宫缩，阴道检查宫口开1 cm（宫颈消退80％），B超提示胎儿生长参数正常但见脐带绕颈（U形压迹）；11月6日突发胎心减速至60次/min（持续1 min），复查宫口开2 cm，考虑急性胎儿宫内窘迫，持续床旁监护产程进展及胎儿情况。11月7日宫缩减弱、宫口停滞于2 cm，经多学科讨论后予催产素引产。11月8日12:00宫口近开全，但胎头下降不明显，胎心消失，产科团队行产钳助娩一死女婴，重1 070 g，胎盘自娩，重200 g。产后患者出血量较多，立即予重组人凝血因子Ⅶa静推，同时输注红细胞、血小板、血浆等治疗。产后4 h患者出现心率加快至160次/min、血压下降至90/54 mmHg、尿量减少，予补液扩容后血压稍回升，同时患者持续高热，查C反应蛋白136 mg/L，降钙素原6.49 µg/L，乳酸3.2 mmol/L，SOFA评分为11分，G试验、GM试验阳性，考虑产妇引产损伤及免疫抑制诱发脓毒症，继续去甲肾上腺素升压，加用两性霉素B胆固醇硫酸酯复合物抗感染，并行床旁连续性肾脏替代治疗（CRRT）清除内毒素和炎症介质。同时经鼻胃管鼻饲伊马替尼联合地塞米松继续诱导治疗。

经综合治疗，患者呼吸氧合功能逐渐改善，肺部渗出吸收（图1B、1C），体温及炎症指标恢复正常（图2A、2B），血小板回升（图2C）。11月11日成功撤除呼吸机并停止CRRT治疗。11月13日起将伊马替尼剂量增至300 mg每日2次。11月15日查胸部CT平扫示双肺弥漫性模糊影（图3A～C）。11月19日复查骨髓象：增生极度低下，未见幼稚细胞，MRD：<5.6×10^−5^，BCR::ABL定量：18.32％。后继续采用伊马替尼+地塞米松“无化疗”方案，具体为伊马替尼400 mg/d，第1～10天，300 mg每日2次，第11～49天；地塞米松15 mg/d，第1～3天，10 mg/d，第4～21天，逐渐减量至第28天停用。

**图2:**
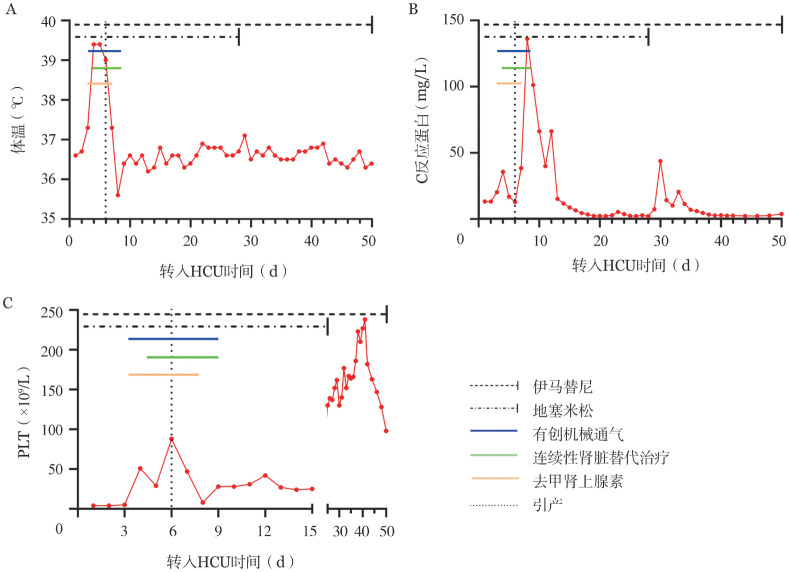
患者从转入血液科重症监护病房（HCU）至出院的体温和实验室指标变化 **A** 体温；**B** C反应蛋白；**C** PLT

12月13日（诱导治疗第41天）患者复查胸部CT示双肺渗出基本完全吸收（图3D～F），12月21日复查骨髓，骨髓细胞形态学示完全缓解，MRD：<2.9×10^−5^，BCR::ABL定量：2.28％，于12月23日从血液ICU步行出院。后在长期口服伊马替尼靶向治疗的基础上先后行IVP方案（去甲氧柔红霉素、长春地辛、地塞米松）、Hyper-CVAD B方案（甲氨蝶呤、阿糖胞苷）巩固化疗各1个疗程，CD19^+^CD22^+^双靶标嵌合抗原受体T细胞（CAR-T）治疗，并于2022年5月行其胞弟来源的半相合异基因外周造血干细胞移植，BCR::ABL融合基因定量逐步下降至不可检测水平。随访截至2025年4月，患者一般情况良好，2023年10月末次复查骨髓，仍处于完全血液学反应（CHR）和完全分子学反应（CMR）状态。

**图3:**
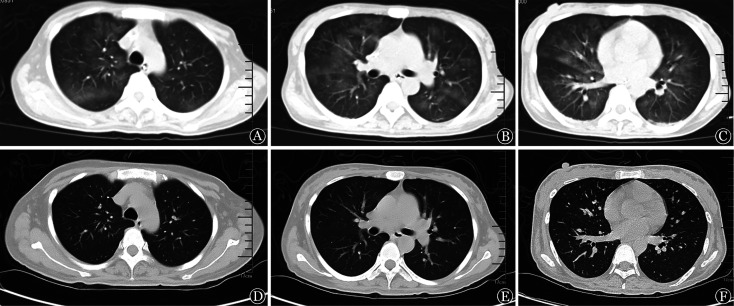
血液科重症监护病房救治过程中胸部CT变化 **A～C** 2021年11月15日胸部CT平扫示双肺弥漫性模糊影；**D～F** 2021年12月13日胸部CT平扫示双肺渗出较前吸收

## 讨论及文献复习

妊娠期急性白血病是一种罕见的产科危重症（发病率约1/10万），其中急性髓系白血病（AML）约占2/3，ALL约占1/3[3]–[4]，ALL好发于儿童及青少年，妊娠期病例相对少见，其症状（如乏力、贫血、血小板减少）易与妊娠反应混淆，常导致诊断延误[5]，因此对不明原因血常规异常者应尽早行骨髓检查[6]。若不及时治疗，可能导致DIC、出血、血栓栓塞甚至孕产妇死亡，并增加早产、胎儿死亡等风险[7]。治疗上需权衡疗效与胎儿安全：孕早期化疗致畸风险高，可能需终止妊娠；孕中晚期可谨慎应用化疗[8]。分娩时机的选择需综合评估孕妇病情及孕周。国内目前缺乏专门指南，需要血液科、产科、新生儿科等多学科团队共同管理，并重视长期随访胎儿发育及母体预后[9]。

妊娠期急性白血病在临床上相对少见，但治疗非常复杂，难度极大，治疗上要同时兼顾产妇和胎儿的安全，需多学科共同协作。妊娠早期流产率高，化疗药物暴露易导致胎儿畸形，故应在足够的支持治疗基础上及时终止妊娠，术后予化疗，可获得与非妊娠患者相似的缓解率及生存率[3],[7],[10]。病情危重者则先予以化疗，待病情缓解后再终止妊娠。妊娠中晚期药物致胎儿畸形的风险明显下降，患者有可能在接受化疗获得缓解的同时娩出健康新生儿，但大部分化疗药物存在致畸作用或对胎儿影响尚不明确，临床医师应充分告知患者及家属，结合患者一般情况及意愿选择合适的治疗方式。临近分娩期化疗可稍许推迟，孕妇状况较差不能耐受产程或手术刺激时，应避免终止妊娠，待末次化疗2～3周后再行考虑[11]–[12]。本文中患者于孕28周诊断为Ph^+^ALL，属妊娠晚期，诱导治疗初期迅速出现危及生命的严重并发症，血流动力学不稳，血小板水平极低，对血小板输注及各种升血小板治疗反应均差，合并有DIC，终止妊娠风险极高，应在HCU高级脏器支持条件下继续诱导治疗，待病情稳定后再终止妊娠。目前国内外对于妊娠期ALL治疗方案的报道相对较少。指南推荐妊娠20周可采用与非妊娠期相同的化疗方案，不宜使用甲氨蝶呤，32周后建议单用泼尼松至临产[13]–[14]。Zhu等[15]报道的3例Ph阴性ALL妊娠患者接受VDCP方案（长春地辛、环磷酰胺、柔红霉素、地塞米松）化疗均取得缓解，其中两例产下健康新生儿。Mainor等[16]报道的1例Ph^+^ALL患者在孕24周时接受伊马替尼联合hyper-CVAD方案（环磷酰胺、地塞米松、阿霉素、长春新碱）化疗，孕30周时接受剖宫产诞下一低体重无畸形新生儿，而产后7周时，孕妇因脓毒症死亡。

随着TKI的问世，Ph^+^ALL的预后得到显著改善，从强化疗方案到弱化疗方案，再到无化疗方案，治疗方案的选择也在不断优化。Ashraf等[17]报道一例妊娠晚期Ph^+^ALL患者，在传统化疗失败后采用一代TKI伊马替尼联合糖皮质激素可有效诱导缓解，并成功分娩健康新生儿。最近的GIMEMA试验证明了无化疗方案对不同患者群体中Ph^+^ALL的有效性。对于老年或不适合移植的患者（44例），三代TKI普纳替尼（45 mg/d，口服8周）联合6周糖皮质激素治疗，24周CHR率为86.4％，CMR率为40.9％，中位无事件生存期为14. 31个月[18]。对于较年轻的成年人（18～60岁，60例），二代TKI达沙替尼（140 mg/d，口服12周）和4周的糖皮质激素治疗，CHR和CMR率分别达到97％和18.3％，5年累积复发率为29.8％，5年无病生存率为47.2％，5年总生存率为56.3％[19]。由此可见，无化疗治疗Ph^+^ALL的安全性及疗效令人满意，可使不适宜行传统化疗的患者获益。

本例产妇妊娠晚期确诊为Ph^+^ALL，诱导治疗初期出现多器官功能障碍、DIC、DAH，脏器功能不能耐受常规化疗，并且化疗后骨髓抑制期感染和出血风险极大，故予TKI联合地塞米松这一“无化疗”方案，在TKI的选择上，综合评估了妊娠安全性和给药途径，伊马替尼作为首个获批的TKI，在慢性髓性白血病妊娠患者中的应用经验较多，胎儿异常发生率约为10％，胎盘渗透性较低，提示其在胎盘形成后可谨慎使用[20]。二代TKI尼洛替尼也有着相对较低的致畸率和胎盘渗透性[21]–[22]，而达沙替尼则具有较高的致畸风险和胎盘渗透性[23]–[24]，二代TKI氟马替尼和三代TKI普纳替尼对发育影响的数据目前尚有限。此外，患者处于气管插管镇静状态，无法经口正常进食，伊马替尼片剂因其可溶于水，适合鼻饲给药而成为最佳选择。产科团队积极配合第一时间及时终止妊娠，并且在HCU层流病房内进行机械通气和血液净化等生命支持治疗，粒细胞缺乏期进行全环境保护，为诱导本病缓解赢得了宝贵时间，患者在诱导治疗第1疗程就取得完全缓解，后在长期口服伊马替尼靶向治疗的基础上先后行IVP方案、Hyper-CVAD B方案巩固化疗，CD19^+^CD22^+^双靶标CAR-T细胞治疗，并行胞弟来源异基因造血干细胞移植，目前仍处于CMR。

在妊娠晚期合并急性白血病中，治疗策略的选择需基于孕周、疾病状态及母胎安全性等多维度评估。随着我国新生儿救治水平的提升，妊娠28周后分娩的新生儿存活率可达90％以上，32周后存活率更高（>95％）[25]。对于妊娠24～32周的患者，临床决策需在充分评估胎儿化疗暴露风险与治疗性早产利弊的基础上进行个体化选择[9]。本例患者转入HCU时病情危重，出现胸闷心悸等循环系统症状，血小板仅为2×10^9^/L且输注效果差，同时合并DIC，表现为全身瘀点瘀斑、血尿及自发性出血。经多学科团队综合评估，认为立即行剖宫产术可能导致难以控制的大出血，严重威胁产妇生命。故决定优先予TKI联合激素控制白血病进展、改善凝血功能，以挽救孕妇生命为首要目标。而对于一般情况稳定、凝血功能经成分输血（如血小板、凝血因子）能够有效纠正的患者，经多学科团队全面评估后，可考虑先行剖宫产终止妊娠，再尽快启动抗白血病治疗[7],[9]。

综上所述，妊娠期急性白血病尤其是Ph^+^ALL在临床十分罕见，母胎存在极大风险，应与患者及家属充分沟通，结合孕周期、孕妇一般情况、胎儿情况等制定个体化治疗方案。对于ICU危重患者，个体化、精细化治疗尤为重要，无化疗治疗为合并有危及生命的并发症的Ph^+^ALL患者提供了安全的疗效确切的新方法，能使更多患者获得更深层的缓解和更长期的生存。
